# Profiling of alternative polyadenylation sites in luminal B breast cancer using the SAPAS method

**DOI:** 10.3892/ijmm.2014.1973

**Published:** 2014-10-20

**Authors:** XINMEI WANG, MINGYUE LI, YINGCHUN YIN, LIANG LI, YUQIAN TAO, DENGGUO CHEN, JIANZHAO LI, HONGMEI HAN, ZHENBO HOU, BAOHUA ZHANG, XINYUN WANG, YU DING, HAIYAN CUI, HENGMING ZHANG

**Affiliations:** 1 Department of Pathology, The Central Hospital of Zibo, Zibo, Shandong 255036, P.R. China; 2 Department of Breast and Thyroid Surgery, The Central Hospital of Zibo, Zibo, Shandong 255036, P.R. China; 3 Department of Neurology, The Central Hospital of Zibo, Zibo, Shandong 255036, P.R. China; 4 Department of Neurology, The First Affiliated Hospital of Zhongshan University, Guangzhou, Guangdong 510080, P.R. China

**Keywords:** profile of alternative adenylation sites, breast cancer, tandem 3′ untranslated region, subtypes in breast cancer, sequencing alternative polyadenylation sites

## Abstract

Breast cancer (BC) is a leading cause of cancer-related mortality in females and is recognized as a molecularly heterogeneous disease. Previous studies have suggested that alternative messenger RNA (mRNA) processing, particularly alternative polyadenylation [poly(A)] (APA), can be a powerful molecular biomarker with prognostic potential. Therefore, in the present study, we profiled APA sites in the luminal B subtype of BC by sequencing APA sites (SAPAS) method, in order to assess the relation of these APA site-switching events to the recognized molecular subtypes of BC, and to discover novel candidate genes and pathways in BC. Through comprehensive analysis, the trend of APA site-switching events in the 3′ untranslated regions (3′UTRs) in the luminal B subtype of BC were found to be the same as that in MCF7 cell lines. Among the genes involved in the events, a significantly greater number of genes was found with shortened 3′UTRs in the samples, which were samples of primary cancer with relatively low proliferation. These findings may provide novel information for the clinical diagnosis and prognosis on a molecular level. Several potential biomarkers with significantly differential tandem 3′UTRs and expression were found and validated. The related biological progresses and pathways involved were partly confirmed by other studies. In conclusion, this study provides new insight into the diagnosis and prognosis of BC from the APA site profile aspect.

## Introduction

Breast cancer (BC) is considered as a leading cause of cancer-related mortality in females, with 232,340 estimated new female cases (~29% of 10 leading cancer types) diagnosed in 2013 (1). Since BC is recognized as a molecularly heterogeneous disease, it is essential to classify the disease into subtypes which are distinguished by pervasive differences in gene expression patterns. Molecular subtypes in BC were first mentioned by Sorlie *et al* (2). The phenotypic diversity observed in BC was mapped to a specific gene expression pattern. Based on the unsupervised hierarchical clustering and microarray results, BC was divided into the luminal A, luminal B, *ERBB2*-associated, basal-like and normal-like subtypes (3). Among these, the luminal A and luminal B subtypes both belong to the luminal subtype, which is the most common subtype (up to 65–70% of female BC cases) (2). Between the 2 subtypes, the luminal B subytpe is characterized as estrogen receptor (ER)/progesterone receptor (PR)-positive and human epidermal growth factor receptor 2 (*HER2*)-positive (ER^+^/PR^+^, HER2^+^), with intermediate expression level of genes in epithelial cells, including *ER*, GATA binding protein 3 (*GATA3*), X-box binding protein (*XBP*), trefoil factor 3 (*TF3*), hepatocyte nuclear factor 3α (*HNF3α*) and estrogen-regulated protein (*LIV-1)* (4,5). The different characteristics between the luminal A and B subtypes are that *HER2* is expressed in the latter subtype, and the latter subtype is also characterized by a high expression of gamma-glutamyl hydrolase (*GGH*), lysosome-associated transmembrane protein 4 beta (*LAPTMB4*), nuclease sensitive element binding protein 1 (*NSEP1*) and cyclin E1 (*CCNE1*). More accurately, the expression of Ki67 is above 14% in the luminal B subtype. Apparent differences in prognosis and response to chemotherapy with respect to the subtypes have been reported in specific patient cohorts (1,2,6).

The 3′ untranslated region (3′UTR) is the section of messenger RNA (mRNA) that immediately follows the translation termination codon and usually is not translated into protein. Through the alternative polyadenylation [poly(A)] (APA) progress, several mRNA isoforms can be produced with variable lengths of 3′UTRs (termed tandem 3′UTRs). The known regulatory role of tandem 3′UTRs is mainly observed in gene expression networks, whereby they influence mRNA stability, transport and translation, generally through the loss and gain of regulatory motifs, such as AU-rich elements (AREs), GU-rich elements and microRNA-binding sites (7–9). While in the human genome, APA phenomena are rather common since more than half of the genes have APA sites (10). Currently there are several methods based on high-throughput sequencing to profile APA sites at the whole-transcriptome level, including RNA-Seq, poly(A) capture, 3P-Seq, sequencing APA sites (SAPAS), direct RNA sequencing (DRS) and poly(A)-tail length profiling by sequencing (PAL-seq) (11–17). Among these, SAPAS provides an unbiased framework for analyzing 3′UTR switching in APA profiling data (18).

In this study, we applied the SAPAS method to generate a comprehensive and high-resolution map of APA sites of human transcripts in one patient diagnosed with the luminal B subtype of BC. Through bioinformatics analysis, we found 153 genes with differential usage of tandem 3′UTRs, which were enriched mainly in focal adhesion and spliceosome pathways and the Wnt receptor signaling pathway, negative regulation of cellular macromolecule biosynthetic process, negative regulation of transcription process and negative regulation of signal transduction process. Moreover, 1,296 differentially expressed genes (DEGs) were discovered to be related in the process of response to endogenous stimuli, response to nutrient levels, cell cycle and cellular chemical homeostasis. Using RT-qPCR approaches, we validated our findings and demonstrated the utilization of the approach to identify APA events. We identifed a number of unannotated poly(A) sites and DEGs to elucidate the possible roles for 3′UTRs in the development of BC and to suggest potential future applications of 3′UTRs in the diagnosis and therapy of BC.

## Materials and methods

### Sample information

**Figure 1:**
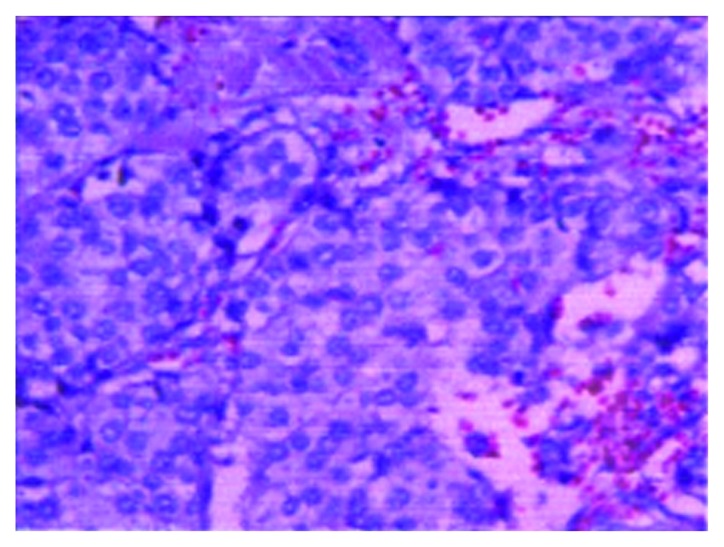
Histological analysis showing the type of breast cancer in one Chinese patient with the luminal B subtype; ×200 magnification.

The mammary normal and cancerous tissues of 9 patients diagnosed with luminal B type BC were obtained through mammary cancer radical operation from the Central Hospital of Zibo, Zibo, China. The normal tissues were mammary cancer-adjacent tissues with no cancer lesions, as shown by a pathological examination. Informed written consent was provided by all patients. Immunohistochemical analysis was performed on paraffin-embedded sections, including markers for ER, PR, HER2/neu (c-erbB-2) and Ki-67. The one patient we selected for SAPAS sequencing was 29 years old and was ER^+++^, PR^+^, c-erbB-2^++^ with a Ki-67 expression of 15%. The tumor size was 4.2×3.5×2.5 cm. According to the above data and histological appearance (Fig. 1), the patient was diagnosed with stage II luminal B type invasive breast ductal carcinoma with focal necrosis and calcification. The detailed immunohistochemical and clinical data of the 9 patients are presented in Table I. This study was approved by the Medicine Ethics Committee of the Central Hospital of Zibo. Informed consent for obtaining the tissue specimens was obtained from all 9 patients.

### RNA extraction

Total RNA of the 18 tissues (normal and cancerous from the 9 patients) was extracted using TRIzol reagent (Invitrogen, Carlsbad, CA, USA) according to the manufacturer’s instructions. A NanoDrop 1000 spectrophotometer (NanoDrop Technologies, Wilmington, DE, USA) and agarose gel electrophoresis were used to detect the amount and quality of the total RNA. RNA purity was assessed by the ratio of absorbance at 260 and 280 nm (A260/A280).

### In vitro transcription-sequencing of APA sites (IVT-SAPAS) library preparation and sequencing

Two libraries from one patient were constructed with the improved method known as IVT-SAPAS as previously described (1). The advantages of IVT-SAPAS are as follows: Firstly, the quantity of the total RNA was reduced from 10 μg to 750 ng, and secondly, using the *in vitro* transcription method, large amounts of RNA were produced from cDNA templates. This improved method is extremely useful when samples are limited. The libraries were then sequenced from the 3′ end with Illumina GAIIx (Illumina Inc., San Diego, CA, USA).

### Raw data analysis

The image files produced by the SAPAS method were transferred into FastQ files through Off-Line Base Caller version 1.9 (Illumina Inc.). The base quality of the raw data for each sample was estimated using FastQC version 0.10.1 (Babraham Institute, Cambridge, UK). Perl scripts were programmed to perform the filtering and trimming of all the reads. Reads with low quality and polyNT (polyNT defined as the fragments with a series of single bases, particularly T) were filtered and the linker 5′-TTTTCTTTTTTCTTTTTT-3′ on the 5′ end of the reads was trimmed. Subsequently, only the long reads (≥25 nt) were obtained. These remaining reads were mapped to the human genome (hg19; downloaded from UCSC genome bioinformatics) (19) (maintained by the University of California Santa Cruz, Santa Cruz, CA, USA) through Bowtie (version 0.12.7; parameters: -v 2 -k 2 -best -q) (20) with bowtie-indexes downloaded from the Center for Bioinformatics and Computational Biology. The uniquely mapped reads were selected to filter reads with internal priming, which refer to the reads mapped to the region within 20 bases downstream of poly(A) cleavage sites containing 12 ‘A’, 5′-AAAAAAAA-3′ or 5′-GAAAA+GAAA+G-3′. These were regarded as disruption sequences since they can bind to primers with their A-rich genomic regions, while not with the poly(A) tail. Perl scripts were used for statistical analysis prior to and following raw data analysis.

### Poly(A) site annotation

According to Tian *et al* (10), all the reads of the 2 samples after internal priming were iteratively clustered as poly(A) cleavage sites. These poly(A) cleavage sites that are located next to each other within 24 nt were further clustered as cleavage clusters. Each cleavage cluster with more than one read was assigned as a poly(A) site. In order to annotate the poly(A) sites, a dataset of all known 3′UTR regions was extracted from the Known Genes database of the UCSC table browser, the detailed procedures were as in the study by Tian *et al* (21), except that the non-coding gene items were kept. With UCSC Known Genes (19) and polyA_DB2 (22), all the poly(A) sites were annotated as known and novel sites. The annotation procedure was the same as in the study by Sun *et al* (23). On the basis of their locations on the genome, all the novel sites were classified as ≤1 knt downstream, 3′UTRs, coding DNA sequences, intergenic sequences, introns and non-coding genes. The poly(A) sites number was calculated.

### Tandem 3′UTR analysis

The tandem poly(A) site was defined with the same conditions as in the study by Tian *et al* (21). The poly(A) sites that overlapped with multiple known 3′UTR regions were not taken into consideration. Thus, genes containing more than one tandem poly(A) site were regarded as genes with tandem 3′UTR. Subsequently, a statistical analysis of tandem APA switch events of these genes, which were expressed in both of the 2 samples, was performed using the linear trend test method. Briefly, the 3′UTR length for each tandem poly(A) site was calculated. A column chain table was then generated as in the study by Sun *et al* (23). Pearson’s correlation co-efficient r, which was in short for tandem APA sites switch index (TSI), was then calculated. The χ^2^ distribution with one degree of freedom was calculated using the following formula: M^2^=(n−1)r^2^. The P-value was then calculated, as was the corresponding false discovery rates (FDRs) rectified by the Benjamini-Hochberg method.

Furthermore, tandem 3′UTR length switching with a FDR cut-off of 1% was considered to be significantly different between the 2 samples. A positive r value indicates a longer tandem 3′UTR in cancer tissue and *vice versa*. More stringently, genes with r<−0.1 and FDR <0.01 were considered as shortened 3′UTR genes, while genes with r>0.1 and FDR <0.01 were regarded as lengthened 3′UTR genes. R software was used to generate a scatter plot illustrating the correlation between them, and the relevant co-efficient was calculated. The number of these 2 types of tandem 3′UTR genes was calculated separately and the χ^2^ test was performed.

### Analysis of DEGs

In addition to test tandem APA switch events, SAPAS can also identify gene expression profiles. Since SAPAS only sequenced the 3′UTRs of mRNAs, the length of each gene could be disregarded when estimating the gene expression level. Thus, the expression level of a gene could be represented by the read number mapped to the corresponding region on the genome. The expression difference of each gene between the 2 samples was assessed by Fisher’s exact test. The P-value was then adjusted through the Benjamini-Hochberg method. Genes with a FDR <0.01 were considered to be DEGs.

### Functional annotation and enrichment analysis

DAVID Bioinformatics Resources 6.7 (24) was used to perform functional annotation and enrichment of the tandem 3′UTR genes and the DEGs separately. Significantly enriched biological process (BP) gene ontology (GO) terms and pathways against a background model of all human transcripts were selected.

### Validation by RT-qPCR analysis

Six genes with switched APA sites [collagen, type I, alpha 2 (*COL1A2*), DEAD (Asp-Glu-Ala-Asp) box helicase 5 (*DDX5*), small nuclear ribonucleoprotein 200 kDa (U5) (*SNRNP200*), catenin, beta interacting protein 1 (*CTNNBIP1*), dishevelled segment polarity protein 3 (*DVL3*) and protein phosphatase, Mg^2+^/Mn^2+^ dependent, 1A (*PPM1A*)] and 5 differentially expressed genes [matrix Gla protein (*MGP*), transforming growth factor β receptor III (*TGFBR3*), insulin-like growth factor 2 (*IGF2*), calcipressin 1 (*RCAN1*) and cyclin D1 (*CCND1*)] were subjected to RT-qPCR to validate the sequencing data (Table II). Total RNA was isolated using TRIzol reagent (Invitrogen) according to the manufacturer’s instructions. For each sample, 1,000 ng of total RNA was then used in reverse transcription reactions using random hexamers and SuperScript^®^ II Reverse Transcriptase (Invitrogen). For each gene, two gene-specific primer sets were designed according to Tian *et al* (21). RT-qPCR was performed using the LightCycler 480 Instrument (Roche Biochemicals, Indianapolis, IN, USA) according to the manufacturer’s instructions. The expression ratios of the shortened region to the lengthened region (cUTR/eUTR, cUTR stands for constitutive UTR, while eUTR stands for extended UTR) were maintained through calculating ΔΔCt values for each gene by normalizing the extended set against the constitutive one. Significantly differential usage of poly(A) sites of genes between samples was detected by the Student’s t-test at a significance level of 0.05. For differentially expressed genes, the relative quantification method was used to measure the levels of the genes in the cancer tissue. *GAPDH* was used as an endogenous control.

## Results

### Sample infomation and sequencing procedure

Total RNA (36.8 and 38.2 μg) was extracted from the normal and cancerous tissue. The OD260/280 value of both samples detected by NanoDrop was 1.88, and the RNA integrity from agarose gel electrophoresis revealed that the total RNA qualified for subsequent procedure. After library construction, the results of Agilent Bioanalyzer 2100 (Agilent Technologies Inc., Santa Clara, CA, USA) revealed that the length of the 2 libraries was approximately 370 bp, located in the normal range of 200–600 bp. All these data showed that the total RNA was properly extracted from the 2 samples and the libraries were regularly constructed.

### Sequencing and data filtering

The 2 libraries were sequenced through the Illumina Genome Analyzer IIx platform. The raw sequencing data were uploaded to the Sequence Read Archive (SRA) database at the National Center for Biotechnology Information (NCBI) and are accessible using the NCBI accession no. SRP041304. In total, 27.4 and 26.6 million raw reads were obtained for the 2 samples (normal and cancerous, respectively). After filtering and trimming, almost 27.3 and 26.5 million reads (99.6%) were remaining. Subsequently, nearly 25.1 and 24.5 million (92.0%) reads were mapped to the human nuclear genome (hg19). Among these, approximately 18.9 million (69.1%) and 18.0 million (67.8%) reads were uniquely mapped. The internal priming reads were then filtered, and 11.5 million (41.8%) and 10.9 million (40.8%) reads could be used to infer transcript cleavage sites and annotate poly(A) sites.

### Poly(A) sites annotation and tandem 3′UTR analysis

**Figure 2:**
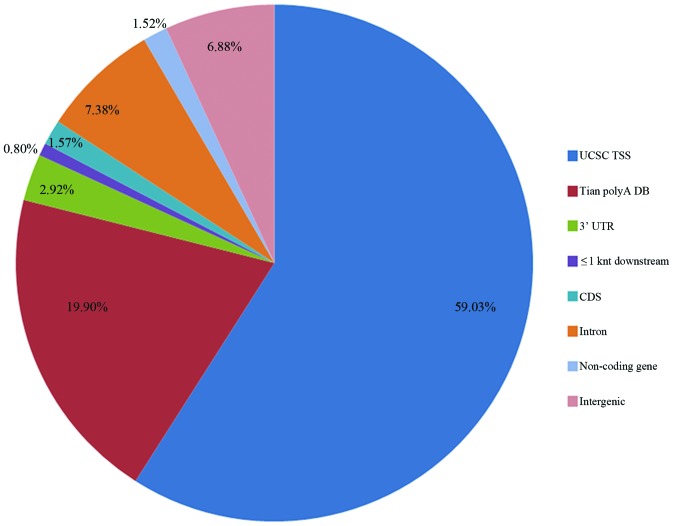
The characteristics of the sequencing alternative polyadenylation (polyA) sites (SAPAS) data. Genomic locations of reads that were uniquely mapped to the nuclear genome after internal priming filtering. Inter, Intergenic; non, non-coding gene; <1 knt, <1 knt downstream; Tian, Tian polyA DB; UCSC, UCSC TTS; CDS, coding DNA sequence.

**Figure 3:**
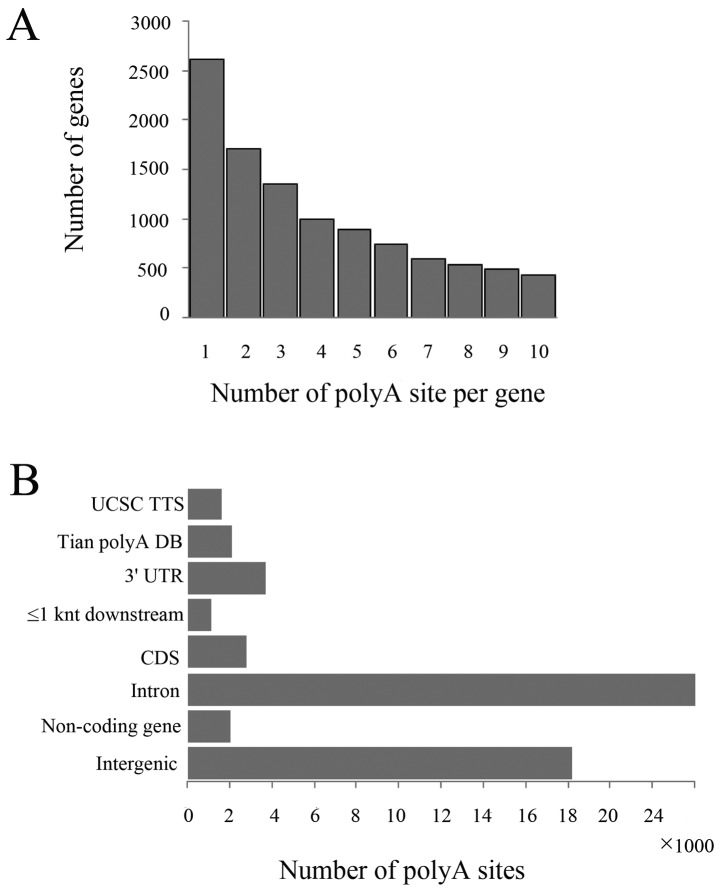
Characteristics of the polyadenylation [poly(A)] site. (A) Distribution of numbers of poly(A) sites per gene. (B) Genomic locations of the poly(A) sites in all genes. Tian, Tian polyA DB; UCSC, UCSC TTS; CDS, coding DNA sequence.

In conclusion, 18,078 UCSC canonical genes with at least one read, which accounted for 66.2% of all canonical genes, were sequenced. The poly(A) sites of 18,448 and 18,970 genes (Table III) were annotated in the normal and cancerous tissue samples. Subsequently, after filtering the poly(A) sites supported with only one read, 16,880 and 17,432 genes with poly(A) sites were obtained for the normal and cancerous tissue, respectively. In all, nearly 78.9% reads were mapped to the region within 24 nt of the known poly(A) sites. This implied that most of the filtered reads produced by this study were mapped to known poly(A) sites in the UCSC transcript ends database and Tian’s database (10). Furthermore, 2.9 and 0.8% of the reads mapped to the 3′UTR region of UCSC canonical gene and 1 kb downstream of the end of the genes (Fig. 2). In addition, in the normal tissue, 16,356 genes (86.2%) were annotated with more than one poly(A) site, while 14,641 genes (77.2%) were annotated with more than 2 poly(A) sites. The distribution of gene number with 1–10 poly(A) sites per gene is shown in Fig. 3A. Among the 206,585 annotated poly(A) sites in the normal tissue, 5.71% of the sites were recorded in the UCSC transcript ends database and polyA_DB2 database and another 51.62, 5.62 and 4.28% of the poly(A) sites in the introns, 3′UTRs and coding sequences (CDSs) from the UCSC canonical genes, respectively. This indicates that a large number of novel poly(A) sites are detected in the mRNAs through the SAPAS method, particularly in lowly expressed mRNAs (Fig. 3B).

**Figure 4:**
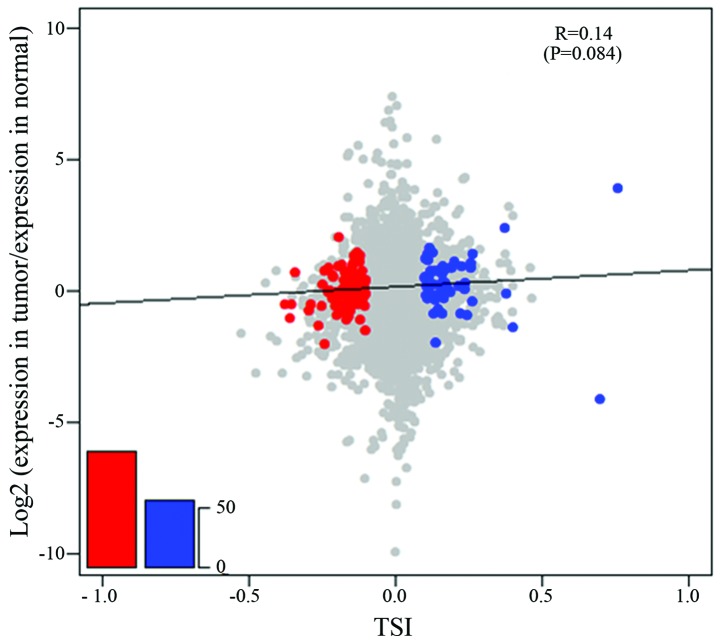
Alternative polyadenylation [poly(A)] (APA), site switching and gene expression levels of paracancerous (normal) and carcinoma tissues. Tandem APA sites switch index (TSI) is plotted against the logarithm of the expression level ratios between the carcinoma and paracancerous tissues. The x-axis denotes TSI, a larger positive value indicates that longer tandem untranslated regions (UTRs) are prone to be used in the carcinoma tissues. Genes with significant switching to longer (blue) or shorter (red) tandem UTRs in carcinoma tissues (FDR <0.01) are colored. The y-axis denotes the logarithm of the expression level of genes from the carcinoma tissues relative to the paracancerous tissues.

After poly(A) sites annotation, a total of 153 genes were indentified to be with a significant difference in tandem 3′UTR length (FDR <0.01). Among these genes, 97 genes (63.4%) had a negative TSI, which suggests that the number of genes with shortened 3′UTRs in the cancerous tissue was slightly more than that of the genes with lengthened 3′UTRs (Fig. 4). The results of statistical analysis using the χ^2^ test (P=9.176×E-04) of the quantitative difference of the shortened and lengthened genes were significant.

### DEGs

Using the reads mapped to the genes, the expression levels in the 2 samples were profiled. The reads supported for each gene were then normalized by the minimum number of the reads after internal priming. Fisher’s exact test and the Benjamini-Hochberg method for rectification were performed to select the DEGs. The genes with a FDR <0.01 and fold-change >3 were regarded as DEGs. Therefore, a total of 1,296 genes was identified as DEGs. Among these, 495 were upregulated by 3-fold in the cancerous tissue compared with those in the normal tissue (FDR <0.01), while 801 genes were downregulated.

### Functional enrichment

The shortened genes in the cancerous tissue were enriched in 39 BP of GO terms primarily associated with negative regulation of macromolecule metabolic process, establishment of RNA localization and RNA transport (FDR <0.05; data not shown). All genes with shortened 3′UTRs were enriched in spliceosome (P<0.05) (Table IV).

A decrease in breast cancer cell growth was associated with the reduction of macromolecule biosynthesis and the induction of apoptosis by global transcriptional profiling (25). There are 11 genes with shortened 3′UTRs which were enriched in the GO category of negative regulation of macromolecule metabolic process (Table V). Among these genes, the tissue inhibitor of metalloproteinase 3 (*TIMP3*), was found to encode proteins which are inhibitors of the matrix metalloproteinases, a group of peptidases involved in the degradation of the extracellular matrix (ECM). The expression of *TIMP3* is induced in response to mitogenic stimulation and this netrin domain-containing protein is localized to the ECM (http://www.ncbi.nlm.nih.gov/nuccore/NG_009117.1). Acording to UniProtKB, complexes with metalloproteinases (such as collagenases) irreversibly inactivates them by binding to their catalytic zinc cofactor. This may form part of a tissue-specific acute response to remodeling stimuli. Studies have demonstrated that *TIMP3*, a mammalian tissue inhibitor, significantly reduces the potential for metastasis for the transfected human BC cell line (26,27). Moreover, mRNAs with shortened 3′UTR often lose the miRNA binding site, which may decrease the expression of the mRNA through miRNA inhibition (20). Thus, switching to the proximal poly(A) site of this gene may enhance the inhibition of ECM degradation and improve the inducement of response to extracellular stimuli, which collectively increases the metastatic potential in the progression of BC. Another gene is v-ski sarcoma viral oncogene homolog (avian) (*SKI*), which also stimulates growth by activating the Wnt signaling pathway (28). Furthermore, 3 genes termed ArfGAP with FG repeats 1 (*AGFG1*), heterogeneous nuclear ribonucleoprotein A2/B1 (*HNRNPA2B1*) and nucleoporin 153 kDa (*NUP153*) were found to be enriched in the establishment of RNA localization and RNA transport. All these genes have ben reported to be related with HIV RNA transport (29–31).

Several genes with shorter 3′UTRs were observed to be enriched in the spliceosome pathway. Among these genes, U2-associated SR140 protein (*SR140*), also known as U2 snRNP-associated SURP domain containing (*U2SURP*), were summarized to be associated with BC by GeneCards (http://www.genecards.org/).

The lengthened genes were enriched in 37 BP of GO terms, which were associated with protein localization and transport (P<0.01; data not shown). Furthermore, these genes were involved in the following 3 pathways: focal adhesion, prostate cancer and Jak-signal transducer and activator of transcription (STAT) signaling pathway (P<0.1) (Table IV). Among the genes enriched in protein localization and transport, trichorhinophalangeal syndrome I (*TRPS1*) was found to encode the TRPS-1 protein, which has been found to be overexpressed in >90% of early- and late-stage BC cases by immunohistochemical analysis (32). However, switching to the distal APA site of this gene may decrease the expression due to miRNA combination. Therefore, further investigations may be required to explain the phenomenon in detail. Strikingly, another gene, termed *STAT6* [interleukin-4 (IL-4)-induced] was enriched in the regulation of transcription. IL-4-induced STAT6 signaling is active in a variety of cell types, including immune cells and cancer cells, and plays an important role in the regulation of gene expression. Zhang *et al* (33) reported that BC cells carrying STAT6 (null) phenotype exhibited increased spontaneous apoptosis compared with those carrying STAT6 (high) phenotype. Using the longer 3′UTR, the role for *STAT6* in the cancerous tissue may be relatively close with the former status.

Moreover, several genes with lengthened 3′UTRs were observed to be enriched in the focal adhesion pathway (Table VI). Among these genes, B-cell lymphoma protein 2α isoform (*Bcl-2*), was discovered to have a significantly lower positive expression rate in the BCs than in the normal tissue (34). Another study showed that the overexpression of the Bcl-2 or Bcl-x(L) associated with the loss of apoptosis in BC cells *in vivo* may account for their metastatic behavior (35). Univariate analysis indicated a decreased Bcl-2 protein expression to be significantly (P=0.0089) associated with a worse disease-free survival (DFS) in BC (36). Real *et al* (37) suggested that an increased activation of the STAT3-Bcl-2 pathway in estrogen receptor-negative metastatic BC cell lines conferred a survival advantage to these cells and contributed to their chemoresistance. Neri *et al* (38) reported that the expression of Bcl-2 improved the prognosis of peritumor lymphovascular invasion (LVI)-positive tumors up to values similar to LVI negative cases, while its absence associated with the presence of LVI resulted in a poor outcome with only 28% of patients alive at 8 years. Therefore, with the lengthened 3′UTR in cancerous tissue, the expression of Bcl-2 may be relatively lower since miRNA binding, which was in accordance with the reported situation above. Furthermore, the lower expression may be associated with the increase in apoptosis, which may lead to the avoidance from the metastatic behavior in BC. From this aspect, the prognosis of BC may be improved with the shortened 3′UTR of this gene. However, detailed and comprehensive experiments and studies are required for further confirmation. Another gene is phosphoinositide-3-kinase, regulatory subunit 1 (*PIK3R1*). It has been demonstrated that Akt is activated by a variety of stimuli, through such growth factor receptors as HER2, in a phosphoinositide-3-OH kinase (PI3K)-dependent manner. A loss of phosphatase and tensin homolog deleted on chromosome 10 (PTEN) function also activates Akt, which is then associated with a worse outcome among BC patients treated with endocrine therapy (39). These data suggest that the *PIK3R1* gene with a longer 3′UTR is involved in the associatino between endogenous and extracellular stimuli and the prognosis of BC.

The upregulated genes were mainly enriched in mitotic cell cycle, nuclear division, mitosis M phase of mitotic cell cycle, cell cycle phase, organelle fission, M phase, cell cycle process, cell cycle, cell division and chromosome segregation (FDR <0.01). The GO categories of response to endogenous and extracellular stimuli, including steroid hormones (such as glucocorticoids, corticosteroids and estrogen) and nutrient levels (organic substances and vitamins), chemical homeostasis, locomotory behavior, cell-cell signaling, regulation of cell proliferation, neuronal differentiation, pattern specification process, cellular component morphogenesis, cell projection morphogenesis were enriched among the downregulated genes (FDR <0.05).

### Validation by RT-qPCR

**Figure 5:**
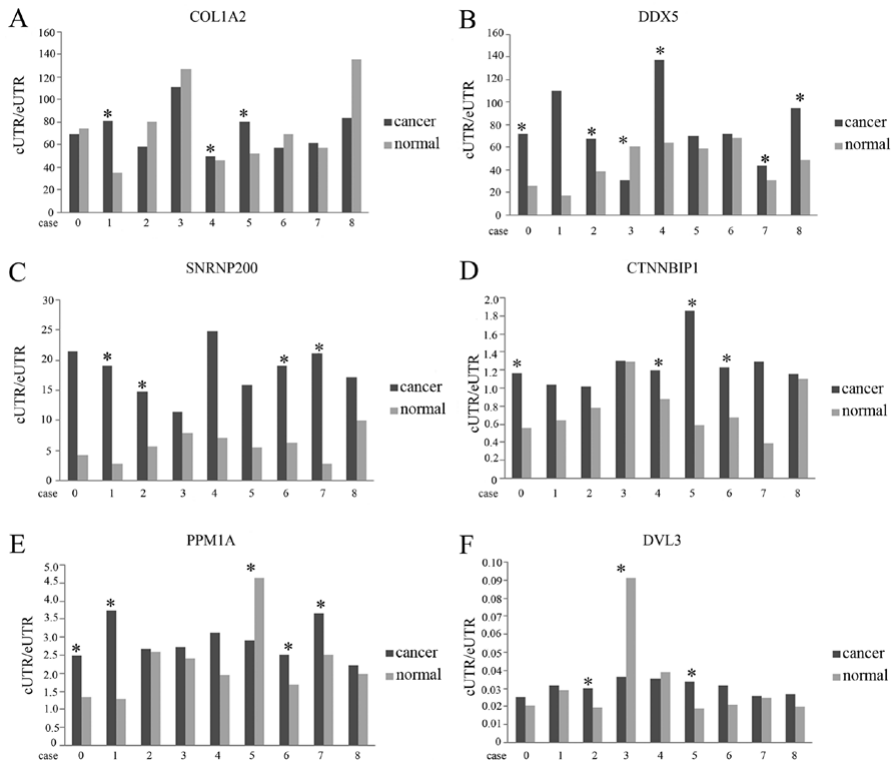
Relative changes in expression level of tandem 3′ untranslated regions (3′UTR) genes in 9 patients detected by RT-qPCR. (A) *COL1A2*. (B) *DDX5*. (C) *SNRNP200*. (D) *CTNNBIP1*. (E) *PPM1A*. (F) *DVL3*. cUTR/eUTR, constitutive UTR/extended UTR, the expression ratios of the shortened region to the lengthened region. ^*^P<0.05.

For further confirmation of the poly(A) site-switching regulation of the tandem 3′UTR genes, 6 genes with switched APA sites (*COL1A2*, *DDX5*, *SNRNP200*, *CTNNBIP1*, *DVL3* and *PPM1A)* (Table VII) were subjected to RT-qPCR in the sequenced patient and an additional 8 patients. Among the 6 genes, *SNRNP200* and *DDX5* were enriched in the spliceosome. Another 2 genes (*PPM1A* and *DVL3*) were enriched in the Wnt receptor signaling pathway. According to UniProtKB, *DVL3* may play a role in the signal transduction pathway mediated by multiple Wnt genes. Furthermore, *CTNNBIP1* is involved in the negative regulation of macromolecule metabolic process, regulation of transcription, Wnt receptor signaling pathway, negative regulation of transcription from RNA polymerase II promoter, negative regulation of signal transduction. The lengthened gene, *COL1A2*, was enriched in focal adhesion. Three transcripts, resulting from the use of APA signals, have been identified for this gene (http://www.ncbi.nlm.nih.gov/gene/1278). It has been demonstrated that the COOH-terminal fragment of procollagen type I (C3) is produced in tissues with high synthesis of collagen I, such as in BC stroma and in bone (40). However, this represented a paradox between the lower expression of certain genes, which tended to use longer 3′UTR, with the high synthesis in BC. Further investigation is required to reconcile this paradox. All these candidate genes with switched APA sites were validated in the sequenced patients. Furthermore, for the shortened genes, 2 genes (*SNRNP200* and *CTNNBIP1*) tended to use shortened 3′UTR transcripts in all the 9 patients, 3 genes (*DDX5*, *PPM1A* and *DVL3*) tended to be the same as the sequenced patients in 7–8 patients. Moreover, in the lengthened genes, *COL1A2* was validated in 5 patients (Fig. 5).

**Figure 6:**
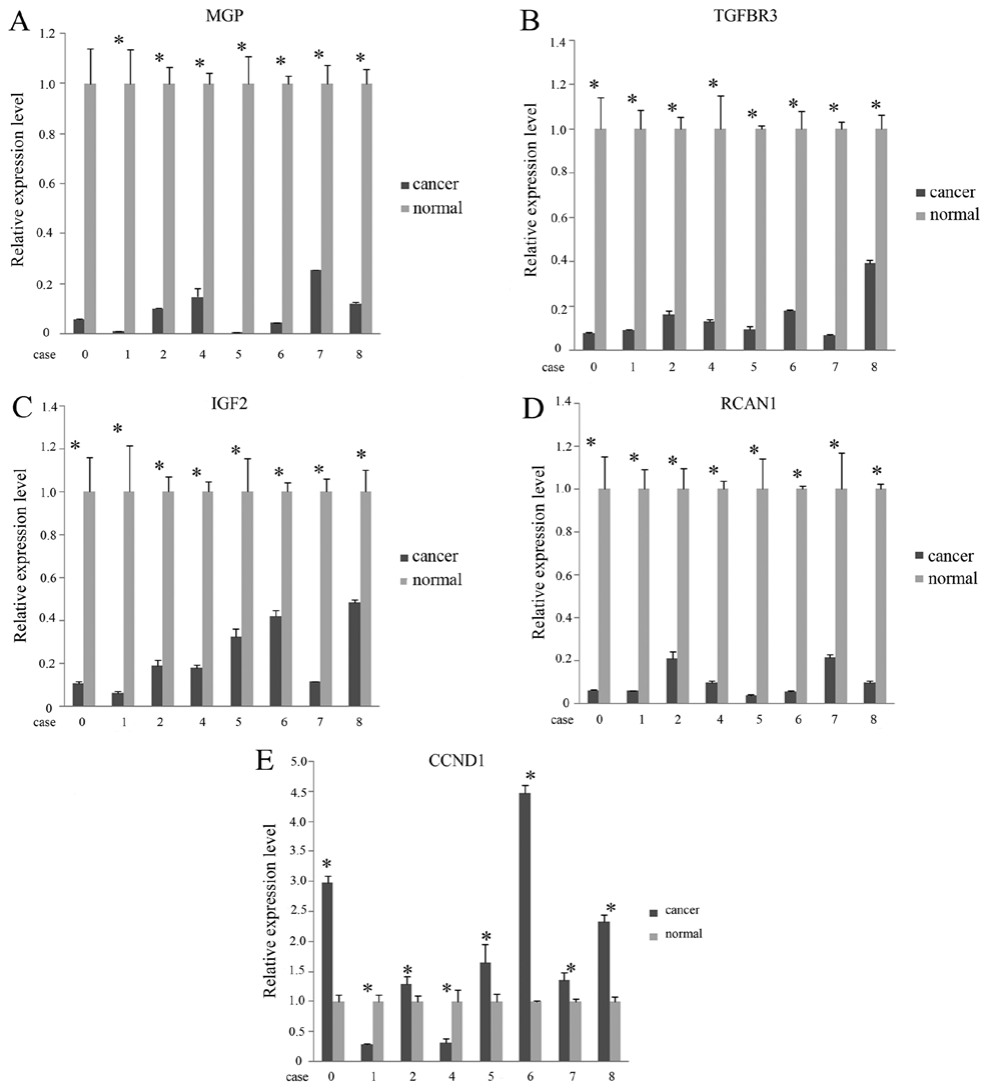
Relative changes in expression level of differentially expressed genes in 9 patients detected by RT-qPCR. (A) *MGP*. (B) *TGFBR3*. (C) *IGF2*. (D) *RCAN1*. (E) *CCND1*. cUTR/eUTR, constitutive UTR/extended UTR, the expression ratios of the shortened region to the lengthened region. ^*^P<0.05.

Five DEGs (*MGP*, *TGFBR3*, *IGF2*, *RCAN1* and *CCND1*) were selected to be confirmed by RT-qPCR in 9 patients. The 4 upregulated genes in cancerous tissue (*MGP* precursor response to hormone stimulus, response to nutrient), *TGFBR3*, *IGF2* isoform 1, *RCAN1* isoform c were enriched in response to hormone stimulus, nutrient and wounding, regulation of cell proliferation, cell morphogenesis, homeostatic process and cell-cell signaling. The downregulated gene, *CCND1*, was enriched in the mitotic cell cycle. As for the 5 DEGs, the regulation pattern detected by RT-qPCR in the sequenced patient revealed a consistent tendency with the one by SAPAS. Further, the 4 upregulated genes tended to be significantly upregulated in the cancerous tissue in 8 patients (P<0.05; t-test ), while the one downregulated gene tended to be significantly downregulated in 6 patients (Fig. 6).

## Discussion

Although BC cell lines have been used widely to investigate pathobiology and new therapies in the disease, and one of the major benefits is that they offer an infinite supply of a relatively homogeneous cell population that is capable of self-replication in standard cell culture medium (41), at the genomic level, it is uncertain as to how well cell line subtypes faithfully represent tumor subtype counterparts. In a previous study, gene expression patterns in 2 tumor samples from the same individual were almost always more similar to each other than either was to any other sample (42). Taken together, it is essential to further investigate the transcriptional profiles in BC *in vivo*. In the present study, we obtained the comprehensive profiles of APA sites in the luminal B subtype of BC using the SAPAS method.

The study by Singh *et al* (43) suggested that alternative mRNA processing, particularly APA, can be a powerful molecular biomarker with prognostic potential. By the global shortening of 3′UTRs *in vitro* and *in vivo* (16,43), the 3′UTRs show distinct features in primary cancer samples. With shortened 3′UTRs, functional consequences have been produced by genes, which has led to greater mRNA stability and increased protein output (8). Through forcing the expression of shorter 3′UTR isoforms, phenotypic consequences were observed, which suggests that 3′UTR shortening is associated with cell proliferation, including T-cell activation or early embryogenesis (7,44). Furthermore, unlike primary tumors, there are significantly more genes with lengthened 3′UTRs in a metastatic cell line (15). These discoveries suggest that there may be a dynamic deregulation of APA during the life cycle of cancer cells. In this study, we found a significantly greater number of genes with shortened 3′UTRs in the samples with luminal B type BC, which belonged to primary cancer with a relatively low proliferation. Thus, it can be deduced that the global profile of alternative 3′UTRs may be used to classify different tumor stages in the process of the cancer.

Through detailed functional enrichment analysis, we discovered that the global functional enrichment indicates the importance of 3′UTR switching in the spliceosome and focal adhesion pathway, as well as the negative regulation of macromolecule metabolic process, regulation of transcription, the Wnt receptor signaling pathway and negative regulation of signal transduction. It has been identified that the spliceosome and Wnt receptor signaling are among the most commonly identified KEGG pathways and GO processes (45). Moreover, WNT signaling has been reported to be enhanced and may contribute to the proliferation of human breast tumor cells (46). The study by Lamb *et al* (47) demonstrated that WNT pathway activation is significantly higher in populations enriched for BC stem-like cells (BCSCs), while populations enriched for normal stem-like cells have lower levels of WNT signaling. In addition, global transcriptional profiling revealed that a decrease in cell growth is associated with the reduction of macromolecule biosynthesis and the induction of apoptosis (25). These data demonstrate that the related biological progresses and pathways involved in 3′UTR switching are partly confirmed.

A total of 495 upregulated genes were discovered to be involved in the mitotic cell cycle, nuclear division, mitosis M phase of mitotic cell cycle, cell cycle phase, organelle fission, M phase, cell cycle process, cell cycle, cell division and chromosome segregation. A previous study revealed that chromosome 2 open reading frame 40 (*C2ORF40*), which acts as a tumor suppressor gene in BC pathogenesis and progression, functions at the G2/M phase by downregulation of mitotic gene expression (48). Another study demonstrated the ability of dimethyl melaleucate (DMM), a pentacyclic triterpene to exhibit cell cycle arrest at the G0/G1 phase by the downregulation of cyclin D1 through PI3K/AKT inhibition (49). Both studies confirmed the upregulation of mitotic cell cycle genes in BC formation. A clinical study found that BC is caused by a homeostatic imbalance of cell division (50). The GO categories of response to endogenous and extracellular stimuli (including steroid hormones, such as glucocorticoids, corticosteroids and estrogen) and nutrient levels (organic substances and vitamins), chemical homeostasis, locomotory behavior, cell-cell signaling, regulation of cell proliferation, neuronal differentiation, pattern specification process, cellular component morphogenesis, cell projection morphogenesis were enriched among the 801 downregulated genes. Among these pathways and biological progresses, response to hormone stimuli, response to endogenous stimuli and response to steroid hormone stimuli were considered to play an important role in the occurrence and development of breast invasive ductal carcinoma (IDC) (51). These data suggest that apart from the processes and pathways identifed by previous studies, novel and correlative functions involved in differential expression were discovered.

In conclusion, the trend of APA site-switching events in 3′UTRs in the luminal B subtype of BC were found to be the same as those in MCF7 cell lines despite of less prevalence. From the aspect of the APA profile, it can suggest that the luminal B subtype of BC is not highly proliferative *in vivo*, which may provide novel information on the clinical diagnosis and prognosis on a molecular level. Several potential biomarkers with significantly differential tandem 3′UTRs and expression levels were found and validated. The related biological progresses and pathways involved were partially confirmed by a previous study (15). Nevertheless, further, more detailed investigations and research are required to fully elucidate the association between APA profiles and BC tumorigenesis.

## Figures and Tables

**Table I:** Clinical and immunohistochemical characteristics of the 9 patients with luminal B type BC.

Case	Age (years)	ER	PR	c-erbB-2	Ki-67 (%)	Stage
Case 0^a^	29	+++	+	++	15	II
Case 1^b^	36	+	+	+++	60	II–III
Case 2^b^	58	+	−	++	40	III
Case 3^b^	50	++	+	++	70	I
Case 4^b^	30	+	+	+++	30	III
Case 5^b^	44	++	++	0	55	II–III
Case 6^b^	46	++	+++	+	20	II–III
Case 7^b^	41	+	+	+++	80	III
Case 8^b^	47	+	+++	+++	25	III

a Samples for SAPAS;

b Samples for RT-qPCR validation.

BC, breast cancer; ER, estrogen receptor; PR, progesterone receptor; SAPAS, sequencing alternative polyadenylation sites.

**Table II:** Primers used in RT-qPCR.

Genes	Forward primer	Reverse primer
RT-qPCR primers for genes with switched APA sites		
*COL1A2*-S	ATCTACTTGCTTAAATTGTGGGC	GGTTGACATTTTCCATAACAGGT
*COL1A2*-L	GCCAGTCTCATTTTCATCTTCTT	ATGCTTTATTTCATTTTTTTCACAA
*DDX5*-S	ACATAAAGCAAGTGAGCGACC	CCTCTACCCCTGGAACGAC
*DDX5*-L	CTTTCGGGGGAGAGGGTA	CAGGCTGGACACAACACACAT
*SNRNP200*-S	TTTTGGGTAAAGGAGAGTTGAGC	AAGGGAAAGGAAGTGGAGGTAG
*SNRNP200*-L	ACTACCACAAGAACCAACACTGAG	GGGTCACATCCAGCTAGTACATTT
*CTNNBIP1*-S	ACTCAGTGGGGCTGGCAT	AAGGTTTCTGTTGGTCAAGATTTA
*CTNNBIP1*-L	GCCCCCTCTTTGTAGCTCCT	CAGCAACACTTTGACTTTTCCTCT
*PPM1A*-S	TGTGTTTGGACTTGGGGTT	AGTTAAATGAAGGGACTGGCT
*PPM1A*-L	CAACCACCACCAATGCACA	TAGTCAAGGGATAACCAGGTAAGA
*DVL3*-S	TGTGGATGTGATGTGAGCAGG	GACAAAGTAAAAAAGACGGACGG
*DVL3*-L	GTAGTCGCCTCCAATAGCCAT	GGTTAGTAGGGTTAGGGGTCTGAA
RT-qPCR primers for differentially expressed genes		
*MGP*	CCTTCATATCCCCTCAGCAGA	GCAGCATTGTATCCATAAACCA
*TGFBR3*	CCTTGGGGACAGTAGTGGTTG	GTGATGTTTCCGTGGGGCT
*IGF2*	CATCGTTGAGGAGTGCTGTTTC	ACTGCTTCCAGGTGTCATATTG
*RCAN1*	GATGCGACCCCAGTCATAAAC	TTCCTCTTCTTCCTCCTTCTCT
*CCND1*	GCATCTACACCGACAACTCCA	TTGTTCTCCTCCGCCTCTG

S, primer for shortened region of 3′UTRs; L, primer for lengthened region of 3′UTRs; APA, alternative polyadenylation.

**Table III:** Summary statistics of SAPAS data from Illumina GAIIx sequencing.

	s1	s2
Raw reads	27,392,854	26,620,195
Clean reads, n (%)	27,277,251 (99.6)	26,510,992 (99.6)
Mapped to genome, n (%)	25,188,415 (92.0)	24,492,603 (92.0)
Uniquely mapped to genome, n (%)	18,928,605 (69.1)	18,042,088 (67.8)
Mapped to nuclear genome, n (%)	16,684,903 (60.9)	15,181,455 (57.0)
Passed internal priming filter, n (%)	11,452,320 (41.8)	10,866,946 (40.8)
Genes sampled by reads	18,448	18,970
Poly(A) sites	206,585	263,911
Known poly(A) sites sampled	28,651	29,338
Putative novel poly(A) sites	177,934	234,573
Genes sampled by poly(A) sites	16,880	17,432

SAPAS, sequencing alternative polyadenylation [poly(A)] sites.

**Table IV:** Enrichment of genes with tandem 3′UTR involved in various important GO and KEGG pathways.

GO category	ID	Name	Counts	Fold	P-value
Shortened genes (n=97)					
KEGG_PATHWAY	hsa03040	Spliceosome	4	6.46	2.06E-02
GOTERM_BP_FAT	GO:0016055	Wnt receptor signaling pathway	4	5.98	2.82E-02
Lengthened genes (n=56)					
KEGG_PATHWAY	hsa04510	Focal adhesion	4	5.95	2.33E-02
KEGG_PATHWAY	hsa05215	Prostate cancer	3	10.08	3.10E-02
KEGG_PATHWAY	hsa04630	Jak-STAT signaling pathway	3	5.79	8.38E-02

3′UTR, 3′ untranslated region; GO, gene ontology.

**Table V:** Eleven genes enriched in GO terms associated with the negative regulation of macromolecule metabolic process.

ucscID	Gene symbol	Description	Pearson’s r
uc010wbb.1	*PHF12*	PHD finger protein 12 isoform 1	−0.18
uc011ebr.1	*HEY2*	Hairy/enhancer-of-split related with YRPW motif	−0.36
uc001aqk.1	*CTNNBIP1*	Catenin, β interacting protein 1	−0.21
uc010ikt.2	*SNCA*	α-synuclein isoform NACP112	−0.38
uc003anb.2	*TIMP3*	Tissue inhibitor of metalloproteinase 3	−0.12
uc003geu.1	*MXD4*	MAD4	−0.19
uc001aja.3	*SKI*	v-ski sarcoma viral oncogene homolog	−0.17
uc003oql.2	*FOXP4*	Forkhead box P4 isoform 1	−0.23
uc003bqf.2	*BHLHE40*	Basic helix-loop-helix family, member e40	−0.25
uc002vyg.2	*ASB1*	Ankyrin repeat and SOCS box-containing protein	−0.12
uc003ccx.3	*THRB*	Thyroid hormone receptor, β	−0.24

GO, gene ontology.

**Table VI:** Four genes enriched in GO terms associated with focal adhesion.

ucscID	Gene symbol	Description	Pearson’s r
uc002lit.1	*Bcl-2*	B-cell lymphoma protein 2α isoform	0.21
uc003ung.1	*COL1A2*	α2 type I collagen precursor	0.18
uc011cir.1	*PDGFC*	Platelet-derived growth factor C precursor	0.16
uc003jva.2	*PIK3R1*	Phosphoinositide-3-kinase, regulatory subunit 1	0.14

GO, gene ontology.

**Table VII:** Six tandem 3′UTR genes validated by qRT-PCR.

ucscID	Gene symbol	Description	Pearson_r
uc003ung.1	COL1A2	α2 type I collagen precursor	0.18
uc010deh.2	DDX5	DEAD (Asp-Glu-Ala-Asp) box polypeptide 5	−0.14
uc002svt.2	SNRNP200	Activating signal cointegrator 1 complex subunit	−0.16
uc001aqk.1	CTNNBIP1	Catenin, β interacting protein 1	−0.21
uc001xew.3	PPM1A	Protein phosphatase 1A isoform 3	−0.13
uc003fms.2	DVL3	Dishevelled 3	−0.21

3′UTR, 3′ untranslated region.
